# Expression of Scavenger receptor A on antigen presenting cells is important for CD4^+^ T-cells proliferation in EAE mouse model

**DOI:** 10.1186/1742-2094-9-120

**Published:** 2012-06-07

**Authors:** Hilit Levy-Barazany, Dan Frenkel

**Affiliations:** 1 Department of Neurobiology, George S. Wise Faculty of Life Sciences, Sherman Building, Room 424, Tel Aviv, 69978, Israel

**Keywords:** Scavenger receptor A, SRA, CD4^+^ T-cell, EAE, Multiple sclerosis, Macrophage, APC, Microglia, Astrocyte

## Abstract

**Background:**

Multiple sclerosis (MS) is an autoimmune disease of the central nervous system (CNS) characterized by damage to the neuronal myelin sheath. One of the key effectors for inflammatory injury is the antigen-presenting cell (APC). The class A scavenger receptor (SRA), constitutively expressed by APCs, such as macrophages and dendritic cells in peripheral tissues and the CNS, was shown to play a role in the phagocytosis of myelin; however, the role of SRA in the development of experimental autoimmune encephalomyelitis (EAE) and autoimmune reaction in the periphery has not yet been studied.

**Methods:**

We investigated EAE progression in wild-type (WT) vs. SRA^−/−^ mice using clinical score measurements and characterized CNS pathology using staining. Furthermore, we assessed SRA role in mediating anti myelin pro-inflammatory response in cell cultures.

**Results:**

We discovered that EAE progression and CNS demyelination were significantly reduced in SRA^−/−^ mice compared to WT mice. In addition, there was a reduction of infiltrating peripheral immune cells, such as T cells and macrophages, in the CNS lesion of SRA^−/−^ mice, which was associated with reduced astrogliosis. Immunological assessment showed that SRA deficiency resulted in significant reduction of pro-inflammatory cytokines that play a major role in EAE progression, such as IL-2, IFN-gamma, IL-17 and IL-6. Furthermore, we discovered that SRA^−/−^ APCs showed impairments in activation and in their ability to induce pro-inflammatory CD4^+^ T cell proliferation.

**Conclusion:**

Expression of SRA on APCs is important for CD4^+^ T-cells proliferation in EAE mouse model. Further studies of SRA-mediated cellular pathways in APCs may offer useful insights into the development of MS and other autoimmune diseases, providing future avenues for therapeutic intervention.

## Introduction

Multiple sclerosis (MS) is a chronic autoimmune disease of the central nervous system (CNS) characterized by the formation of focal demyelinating plaques in the brain and spinal cord [1-3]. Understanding the mechanisms leading to cumulative neurological disability in MS and developing effective therapeutic strategies to reduce disease progression are major goals in MS research [4-6].

Scavenger receptor A (SRA), also known as CD204, is a trimeric type II transmembrane glycoprotein expressed as two functional splice variants, SRA-I and SRA-II. SRA is constitutively expressed by mononuclear phagocytes, such as macrophages, dendritic cells (DCs), and Kupffer cells in peripheral tissues, and by microglia in the CNS [7,8]. The scavenger receptors have been demonstrated to play an important role in innate immune defense against pathogens by acting as pattern recognition receptors (PRRs) [9]. PRRs are capable of binding a broad range of ligands, including bacterial surface components, chemically modified and endogenous danger molecules released from damaged cells [10].

Recently, it was shown by Cotena *et al.*[11] that SRA could ensure an inflammatory response of the appropriate magnitude via modulation of the activities of pro-inflammatory receptors and production of chemokines. This suggests that SRA might regulate inflammation via macrophage function and consequent in tissue remodeling in pathological conditions. Furthermore, it was shown that SRA deficiency might cause impairment of infarct remodeling, which results in cardiac rupture via insufficient production of interleukin (IL)-10 and enhanced expression of tumor necrosis factor (TNF)α. This suggests that SRA might contribute to the prevention of cardiac rupture after injury [12].

It was reported that SRA, expressed on microglia and macrophages, plays a role in mediating clearance of neurotoxic β-amyloid plaques in CNS tissues in Alzheimer’s disease animal models [13,14]. Along with other receptors of the SR family, such as class B2 scavenger receptors or CD36 [15], RAGE [16] and others [17]. Furthermore, it was found that the expression of these microglial Aβ phagocytic receptors decreased significantly in transgenic AD mice and, thereby, promoted Aβ accumulation and contributed to neurodegeneration [18].

In approximately 85% of cases, MS patients experience a relapsing-remitting form of disease, which is characterized by acute inflammatory demyelinating episodes, and followed by decreased inflammation and partial remyelination. The process of remyelination is insufficient in this disease, and the accumulated load of lesions that fail to remyelinate results in an eventual secondary progressive neurological state [19-21]. Reports using cell culture systems suggested that phagocytosis of myelin by microglia is mediated by SRA and that this process could result in beneficial remyelination, leading to regeneration of axons in the CNS [22-24]. The experimental autoimmune encephalomyelitis (EAE) mouse model is commonly used to study disease pathogenesis and to test new therapeutic approaches. In this model, mice are immunized with myelin antigen and develop different levels of paralysis that can be monitored [25]. Impairment in remyelination was found to accelerate disease progression in the EAE mouse model [26]. Therefore, we hypothesized that SRA deficiency will exacerbate disease progression in the EAE mouse model.

## Methods

### Animals

WT female mice, and mice that are deficient in type I and type II scavenger receptor class A (SRA^−/−^), both from a background of C57BL/6 mice, were purchased from The Jackson Laboratory (Bar Harbor, ME, USA). Mice were kept in a conventional pathogen-free facility at the Tel-Aviv University (TAU), and all experiments were in accordance with TAU guidelines and approved by the TAU animal care committee of animal research.

### Induction of EAE

EAE was induced by subcutaneous immunization of eight-week-old mice in the flanks with 200 μl of an emulsion containing 250 μg of MOG_35–55_ peptide (MEVGWYRSPFSRVVHLYRNGK) and 400 μg of *Mycobacterium tuberculosis* extract H37 Ra (BD Biosciences, Austin, TX, USA) in incomplete Freund’s adjuvant oil. In addition, the animals received intraperitoneal injection of 250 ng of pertussis toxin (List Biological Laboratories, Campbell, CA, USA). on Day 0 and Day 2. Clinical signs of EAE were assessed according to the following score: 0, no disease; 1, loss of tone in the tail; 2, hind limb weakness; 3, hind limb paralysis; 4, hind limb plus forelimb paralysis; 5, moribund state.

### Histopathology analysis

Lumbar spinal cord sections were prepared from EAE-induced WT and SRA^−/−^ mice. Mice were sacrificed by transcardial puncture and saline perfusion, and their spinal cords were rapidly excised and frozen at −70 °C. The spinal cords were cut in 12-μm coronal sections at −20 °C and used for histological examination. Sections were fixed with 4% paraformaldehyde (PFA) in PBS for 10 min, stained for myelin with Luxol Fast Blue (LFB), and for nuclei with hematoxylin (Sigma, St. Louis, MO, USA), and visualized by light microscopy for evaluation of CNS demyelination and cell infiltration.

### Immunohistology analysis

Lumbar spinal cord sections were prepared from EAE-induced WT and SRA^−/−^ mice as described above. Sections were fixed with 4% paraformaldehyde (PFA) in PBS for 10 min and then incubated with blocking solution containing 1% bovine serum albumin (BSA), 8% horse serum and 0.3% Triton X-100, (Sigma) in PBS for 1 h at room temperature. Slides were then incubated with primary antibodies specific for microglia/macrophages (anti-Iba1 (1:1,000) (Wako Pure Chemical Industries, Ltd, Neuss, Germany)), anti-CD11b (1:50) (Serotec) Raleigh, NC, USA), astrocytes (anti-GFAP (1:200) (G9269; Sigma)), and T cells (anti-CD3 (1:30) T-cell receptor (Serotec)) in blocking solution for 1 h at room temperature. Sections were incubated with fluorescent goat anti-rabbit secondary antibody (Alexa Fluor 488; A-11008; Invitrogen, Carlsbad, CA, USA) for 1 h at room temperature and mounted with Vectashield containing DAPI (H-1200; Vector Laboratories, Burlingame, CA, USA). For fluorescent staining using the Odyssey method, sections were incubated with infra-red (IR) dye-conjugated goat anti-rabbit secondary antibody (Li-Cor) for 1 h at room temperature and mounted with aqueous mounting medium containing anti-fading agents (Biomeda, Burlingame, CA, USA). The integrated intensity of section images was quantified in a blinded fashion using the Odyssey application software version 3.0.16, Li-cor Biosciences, Lincoln, Nebraska, USA (21-μm resolution, 0.5-mm offset with highest quality). The Odyssey infrared imaging system allows global imaging of the entire brain slice, which enables more accurate intensity measurements relative to standard immunohistochemical staining approaches. Furthermore, it permits rapid and sensitive identification of protein expression with sufficient scanning resolution [25,27].

### Splenocyte cytokine assay

Splenocytes were isolated from mice 10 days after immunization with 150 μg MOG_35–55_ peptide, plated in 96-well round-bottom plates in Dulbecco’s modified Eagle medium supplemented with 10% FCS, 4 mM l-glutamine, 100 U/mL penicillin, 0.1 mg/mL streptomycin and 1% sodium pyruvate, and maintained at 37 °C in 5% CO2 and 95% relative humidity at a concentration of 1 × 10^6^ cells/well in the presence of 100 μg/ml MOG_35–55_ for either 24 or 48 h. The medium was then collected for cytokine measurements.

### CD4^+^ T cell proliferation and cytokine assay

Splenocytes were isolated from (150 μg) MOG_35–55_ immunized WT mice 10 days after immunization, and the CD4^+^ T cells were positively separated using magnetic beads according to the suggested protocol (BD Biosciences, Austin, TX, USA). To measure proliferation, cells were stained with 4 μM CFSE (V12883; Invitrogen). After 10 min, cells were washed and plated in 96-well round-bottom plates at a concentration of 3 × 10^5^ cells/well, in a serum free medium, containing 0.5% BSA. Splenocytes from naïve WT or SRA^−/−^ mice were added to the culture at a concentration of 7 × 10^5^ cells/well, and stimulated with 20 μg/ml MOG_35–55_. After 24 h, the cells were harvested for proliferation analysis using flow cytometry [28]. The analysis was performed using a FACScan flow cytometer with Cyflogic software, (Turku, Finland). For cytokine measurements the cells where plated at the same concentrations in the presence of 100 μg/ml MOG_35–55_ and after 40 h incubation the cell supernatant was collected.

### ELISAs for cytokines

Quantitative enzyme-linked immunosorbent assay (ELISAs) for IL-2, IL-6, IL-10, IL-17, interferon (IFN)-γ and TNF-α were performed using paired mAbs specific for each of these cytokines following the manufacturer’s recommendations (BD PharMingen Systems, Minneapolis, MN, USA) [28].

### Statistics

GraphPad software (GraphPad Prism Software, Inc., San Diego, CA, USA) was utilized for statistical analysis. Data comparisons were performed using two-tailed unpaired Student's *t*-tests. Differences with *P* < 0.05 were considered significant.

## Results

### SRA deficiency leads to reduction in EAE disease progression

**Figure 1:**
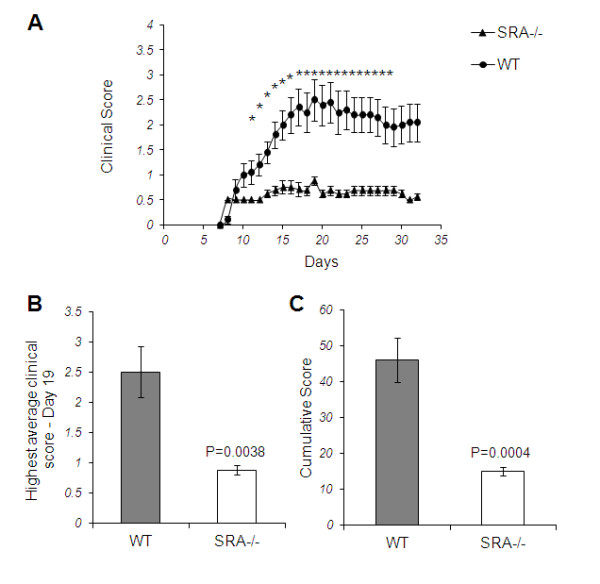
**Disease progression and clinical scores in EAE-induced SRA**^**−/−**^**and WT mice. A**) EAE was induced in SRA^−/−^ and WT mice (n = 9) by subcutaneous immunization with 250 mg MOG_35–55_ peptide emulsified in CFA. Mice were administered 250 ng pertussis toxin i.p. at the time of immunization and 48 h later. Disease scores were measured as follows: 0, no disease; 1, loss of tone in the tail; 2, hind limb weakness; 3, hind limb paralysis; 4, hind limb plus forelimb paralysis; 5, moribund state. Average clinical scores are presented in the graph. **B**) Maximum clinical scores as measured on Day 19. **C**) Cumulative clinical scores of WT and SRA^−/−^ mice. Statistical analysis was performed using Student’s *t*-tests, and results are presented as means ± SEM; *P < 0.01.

To examine the role of SRA in EAE progression, we induced EAE in WT and SRA^−/−^ mice. It is important to indicate that the lack of SRA does not lead to any obvious defects in development or brain pathology in naïve SRA^−/−^ mice. Nine days post-immunization the first clinical signs appeared in both WT and SRA^−/−^ mice (score 0.5) (Figure 1A). Surprisingly, we found that while the clinical score of WT mice increased to a maximum average score of 2.5 ± 0.4 during Days 19 to 21 before remission, the clinical score of SRA^−/−^ mice remained low (0.9 ± 0.08, *P* = 0.0038) through the entire disease course (Figure 1B). In addition, the cumulative score of the SRA^−/−^ mice was significantly lower (67%, *P* = 0.0004) than the cumulative score of the WT mice (Figure 1C).

### Spinal cord lesions are decreased in EAE-induced SRA^−/−^ mice

**Figure 2:**
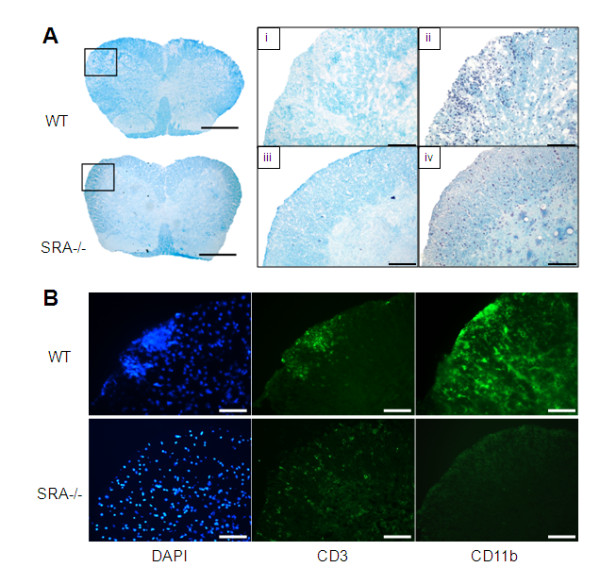
**Histopathology of spinal cord lesions in EAE-induced SRA**^**−/−**^**and WT mice. A**) Representative images of LFB and hematoxylin staining of EAE-induced WT and SRA^−/−^ mice (n = 5). Although LFB staining of WT mice showed demyelination (Ai) in parallel to massive cell infiltration (hematoxylin staining) (Aii), LFB (Aiii) and hematoxylin (Aiv) staining of SRA^−/−^ mice showed reductions or complete loss of demyelination and infiltration. **B**) Representative images of immunohistology staining of the lumbar sections of EAE-induced WT and SRA^−/−^ mice (n = 5). DAPI (nuclear staining), CD3 (a T-cell marker) and CD11b (a microglia/macrophage marker). Original magnification: spinal cord - upper panels – 500 μm; lower panels – 100 μm.

To compare lesion formation in the spinal cords of SRA^−/−^ mice to that of WT mice, we used Luxol Fast Blue (LFB) staining for myelin and hematoxylin staining for cell infiltration. We observed (Figure 2A) in the SRA^−/−^ mice spinal cords significantly less reduction in LFB staining (Aiii) as compared to that of WT mice (Ai), indicating that less demyelination occurred in the SRA^−/−^ mice. Furthermore, using hematoxylin and DAPI staining, we found that the demyelination observed in WT mice (Aii) was correlated with increased mononuclear cell infiltration into the injured area as compared to that observed in SRA^−/−^ mice (Aiv). To analyze the profile of the infiltrating immune cells causing inflammation in the lesion area, we immuno-stained adjacent spinal cord slices for macrophages (CD11b^+^) and T cells (CD3^+^). We found a massive elevation in both macrophages and T cells in the spinal cords of WT mice as compared to that observed in SRA^−/−^ mice (Figure 2B).

**Figure 3:**
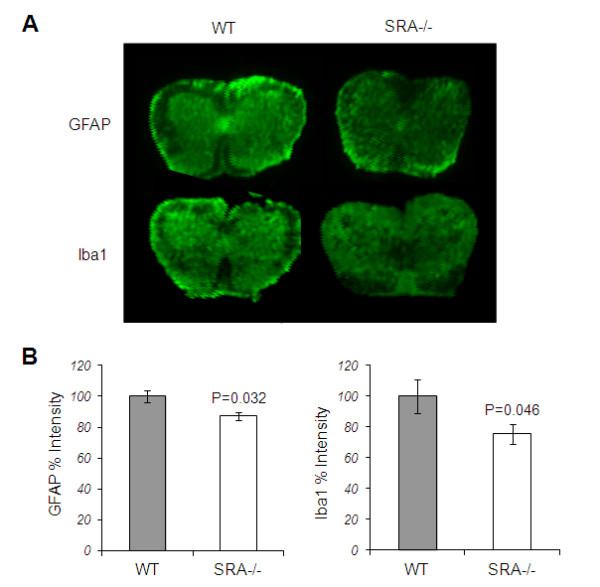
**Immunohistology analysis of spinal cord lesions in EAE-induced SRA**^**−/−**^**and WT mice.** Spinal cord sections from WT and SRA^−/−^ EAE-induced mice (n = 5) were stained, and the fluorescent signals were detected using an Odyssey scanner. (**A**) Representative images of GFAP, an astrocyte marker, and Iba1, a microglia/macrophage marker. (**B**) There was a reduction in signal intensity of GFAP and Iba1 stained SRA^−/−^ sections as compared to WT. Statistical analysis was performed using Student’s *t*-test. Results are presented as means ± SEM.

We further investigated glial activation surrounding spinal cord lesions in EAE-induced mice. We used the Odyssey method to assess fluorescent signal intensities of astrocytes (GFAP^+^) and microglia (Iba1^+^) that are present in the tissue. We found significant reductions of 12% (*P* < 0.05) and 25% (*P* < 0.05) in GFAP^+^ and Iba1^+^ cells, respectively, in SRA^−/−^ mice (Figure 3). These results demonstrate that SRA depletion reduces both CNS lesion formation and immune cell infiltration.

### SRA plays an important role in the induction of pro-inflammatory responses

**Figure 4:**
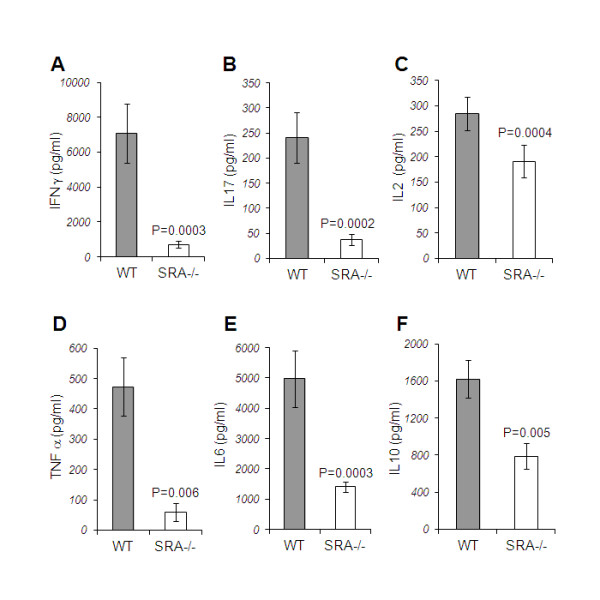
**Reduction in cytokine release following MOG**_**35–55**_**immunization in SRA**^**−/−**^**vs. WT mice.** Spleen cells from MOG_35–55_-immunized WT mice and SRA−/− mice (n = 11) were cultured in a 96-well plate at a concentration of 1 × 10^6^ cells/well in the presence of 100 μg/ml MOG_35-55_. Culture supernatants were collected after 48 h for analysis of (**A**) IFN-γ, (**B**) IL-17, (**C**) IL-2, (**D**) TNF-α (**E**) IL-6 and (**F**) IL-10 by ELISA. SRA−/− splenocytes showed a significant reduction in cytokine production when compared to WT splenocytes. Results are presented as means ± SEM.

Previous studies regarding MS immunopathology have shown that autoreactive pro-inflammatory T cells play an important role in the propagation of CNS tissue injury. A major role in mediating autoimmunity is related to professional antigen presenting cells (APCs), such as macrophages and dendritic cells [29-31]. Interaction between APCs and T cells leads to cytokine-mediated T cell activation and proliferation [32,33]. We further examined the role of SRA in the induction of pro-inflammatory responses against MOG peptide. We measured the levels of cytokines produced by splenocytes isolated from WT and SRA^−/−^ mice following immunization with MOG_35-55_. As shown in Figure 4, we found a massive reduction in pro-inflammatory cytokine production in SRA^−/−^ splenocytes. IFN-γ, a Th1 cytokine, was decreased by 90% (*P* = 0.0003), IL-17, a Th17 cytokine, was decreased by 84.4% (*P* = 0.0002), and IL-2, a cytokine required for T cell proliferation, was decreased by 33% (*P* = 0.0004). In addition, we found significant reductions in cytokines associated with APCs activation, including TNF-α (87.4%, *P* = 0.006) and IL-6 (71.7%, *P* = 0.0003) in SRA^−/−^ splenocytes. We also found that the level of the anti-inflammatory cytokine IL-10 was significantly reduced in SRA^−/−^ splenocytes (51.3%, *P* = 0.005). We further investigated whether T-cells from SRA^−/−^ mice are capable of producing pro-inflammatory cytokines similar to naïve T-cells. We separated CD4^+^ T cells from naïve WT and SRA^−/−^ (Additional file 1: Figure S1) and measured the level of cytokines released following activation with anti-CD3 antibody. We could not detect a difference in levels of IL-2, and pro-inflammatory cytokines: IL-17 and IFN-γ ^+^ T-cells ability to secrete cytokines (Additional file 1: Figure S1). This suggests that while SRA deficiency does not directly impair CD4^+^ T-cells ability to secrete cytokines, it might lead to impairment in their activation with SRA^-/-^ APCs.

### SRA deficiency reduces proliferation and activation of pro-inflammatory CD4^+^ T cells

**Figure 5:**
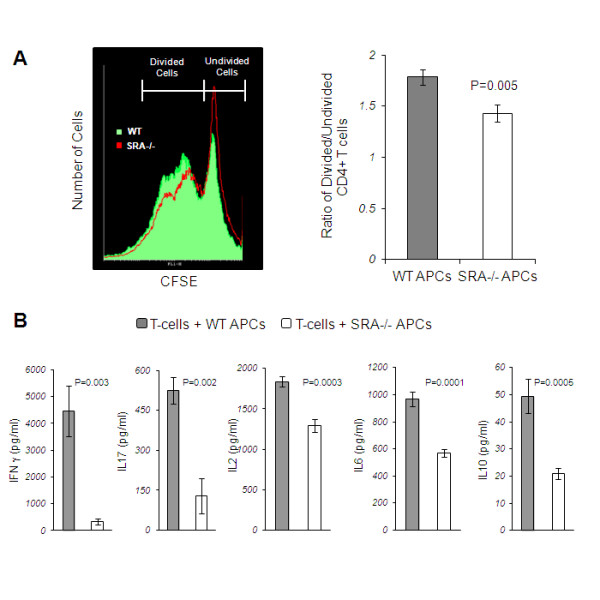
**SRA is essential for myelin-specific CD4**^**+**^**T-cell activation.** WT mice were immunized with 150 μg MOG_35-55_. After 10 days, the CD4^+^ T cells were separated using magnetic beads. The cells were cultured at a concentration of 3 × 10^5^ cells/well and stimulated with 20 μg/ml MOG_35-55_. APCs from naïve WT or SRA^−/−^ mice were added to the cultures at a concentration of 7 × 10^5^ cells/well. (**A**) The CD4^+^ T cells were stained with CFSE and harvested for proliferation analysis using FACS after 24 h. There was a significant reduction in the ratio between divided and undivided CD4^+^ T cells, which were incubated with SRA^−/−^ APCs, as compared to WT APCs. (**B**) Cytokine secretion was measured by ELISA after 40 h. There was a significant reduction in the secretion of IFN-γ, IL-2, IL-17, IL-6 and IL-10 by SRA^−/−^ APCs cultures, as compared to WT cultures. Results are presented as means ± SEM (representative graphs, n = 5 in each of three different experiments.).

We investigated the role of SRA expression on APCs in mediating CD4^+^ T-cell proliferation towards MOG peptide. CD4^+^ T cells from MOG_35-55_-immunized mice were isolated, labeled with CFSE and incubated with APCs which were harvested from non-immunized WT or SRA^−/−^ mice. Following 24 h of incubation, the level of fluorescent signal decreases in proliferating CD4^+^ T cells. We found (Figure 5A) a significant reduction of 19.8% in the ratio between divided and undivided CD4^+^ T cells, that were incubated with WT APCs vs SRA^−/−^ APCs (1.78 ± 0.077 vs 1.43 ±0.083 respectively, *P* = 0.005). In addition, we found a marked reduction in the levels of secreted pro-inflammatory CD4^+^ T-cell cytokines such as IFN-γ, a Th1 cytokine, (92.3%, *P* = 0.03) and IL-17, a Th17 cytokine (75.2%, *P* = 0.002), as well as a 29.6% reduction in IL-2 (*P* = 0.0003). Furthermore, we detected a 41.3% reduction in IL-6, an APC cytokine (*P* = 0.0001), and a 23.2% reduction in the anti-inflammatory cytokine IL-10 (*P* = 0.0006) (Figure 5B). These results indicate that the expression of SRA on the surface of APCs is essential for proliferation and activation of pro-inflammatory CD4^+^ T cells.

## Discussion

In this work we investigated the role of SRA in the propagation of disease in the EAE animal model. We showed that SRA expression is essential for activation and proliferation of pro-inflammatory CD4^+^ T cells that are important for disease initiation.

We discovered that SRA^−/−^ mice immunized with MOG_35-55_ were significantly less susceptible to EAE induction and showed a marked reduction in clinical scores when compared to MOG_35-55_-immunized WT mice. Furthermore, fewer infiltrated T cells (CD3^+^) were found in the spinal cords of EAE-induced SRA^−/−^ mice than in those of WT mice, and significant reductions were observed in the numbers of macrophages (CD11b^+^) and astrocytes (GFAP) surrounding the infiltrated T cells in SRA^−/−^ mice. These findings suggest that SRA could play an important role in EAE disease initiation in the periphery.

The APC-T-cell interaction is a well-known key effector of inflammatory injury in MS. A blockade of this interaction was shown to be effective in ameliorating EAE [34,35]. We demonstrated that SRA-deficient APCs had an impaired ability to drive to pro-inflammatory T-cell proliferation in response to MOG_35-55_ and to drive production of cytokines that are known to play crucial roles in EAE, including IL-2, IL-17, IFN-γ, TNF-α and IL-6. In contrast, we observed a significant reduction in the anti-inflammatory cytokine IL-10, which was previously suggested to ameliorate EAE. These results may suggest that the reduction in pro-inflammatory Th1 and Th17 cell proliferation is due to impaired APC-T-cell interactions and not due to proliferation of anti-inflammatory Th2 cells.

Nicoletti *et al.*[36] have shown that spleen cells of SRA^−/−^ mice immunized with maleylated murine serum albumin (Mal-MSA) exhibit significantly reduced proliferative responses to the antigen when compared to those observed in WT mice. Furthermore, addition of WT APCs rescued proliferative responses to Mal-MSA in splenocyte cultures derived from immunized SRA^−/−^ mice. In addition, SRA has been suggested to play a role in the activation of macrophages in autoimmune diseases [37], such as type 1 diabetes [38] and atherosclerosis [39-41]. In atherosclerosis, SRA has been implicated in mediating macrophage uptake of oxidized-low-density lipoprotein (ox-LDL) [39-41]. Nevertheless, the role of SRA in activation of adaptive immune response was not fully understood. Here we investigated whether the possible mechanism by which SRA mediates disease initiation is through interaction between MOG_35-55_-specific CD4^+^ T cells and APCs. We isolated APCs from spleens of naïve SRA^−/−^ or WT mice and tested their ability to induce proliferation and activation of MOG_35-55_-specific CD4^+^ T cells. These experiments demonstrate that APCs isolated from SRA^−/−^ mice cause significantly less activation and proliferation of CD4^+^ T cells due to a reduction in the levels of pro-inflammatory cytokines produced when compared to APCs isolated from WT mice. Furthermore, we found a reduction in the Th2 cytokine IL-10. These results indicate that SRA plays an important role in the interaction between APCs and CD4^+^ T cells. Therefore, the reduction in the proliferation of CD4^+^ T cells observed in the presence of SRA^−/−^ APCs may relate to the impaired activation of SRA^−/−^ APCs as indicated by reduced levels of TNF-α and IL-6 and not to the secretion of anti-inflammatory cytokines such as IL-10. In addition, according to our results direct activation of CD4^+^ T-cells form SRA^−/−^ using anti CD3 antibody showed that those cells are able to secrete cytokines as WT CD4^+^ T-cells. Moreover, it was shown as well by Haworth *et al.*[42] that SRA^−/−^ intra-peritoneal macrophages, which were injected with LPS, release an increased amount of pro-inflammatory cytokines – TNF-α and IL-6 as compared to WT, suggesting that the SRA^−/−^ macrophage can be activated. Therefore, our results suggest that SRA deficiency may affect APC and CD4-T-cell interaction leading to reduction of antigen specific CD4^+^ T-cell proliferation.

The role of SRA in mediating the proliferation of CD8^+^ T cells was previously reported. SRA was suggested to mediate OVA-specific CD8^+^ T-cell proliferation in a cancer model, and SRA deficiency was found to promote the expansion and activation of cancer-specific CD8^+^ T lymphocytes [43]; however, it should be noted that this previous study investigated the effect of SRA on OVA-specific cytotoxic CD8^+^ T cells in a cancer model and not on endogenous CD4^+^ T cells in an autoimmune model as demonstrated in the current study.

Here we suggest that SRA may play a dual role in EAE progression. The interaction between SRA^+^ APCs to CD4^+^ T-cell is important for disease initiation against myelin antigen. However, as reported before [22-24] in cell culture, phagocytosis of myelin by microglia is mediated by SRA and could result in beneficial remyelination, leading to regeneration of axons in the CNS. Thus, targeting specificly SRA expression in the periphery may reduce disease initiation and allow recovery mechanisms in the brain. The scavenger receptor family contains several different receptors that might also be important for APC activation in response to different antigens. Although we have demonstrated the important role of SRA-I/II in mediating autoimmune responses in EAE, other scavenger receptors might be found to be key players in MS and other autoimmune diseases. Therefore, targeting the activity of different scavenger receptors may prove useful in future diagnostic and therapeutic interventions in autoimmune diseases, such as MS.

## Conclusions

In this study, we have shown the essential role of SRA in mediating pro-inflammatory CD4^+^ T-cell activation and proliferation, which is crucial for disease initiation and progression in EAE. Further studies of SRA-mediated cellular pathways in APCs may provide useful insights into the development of MS and other autoimmune diseases, providing future avenues for therapeutic intervention.

## Abbreviations

APC: antigen presenting cell; BSA: bovine serum albumin; CNS: central nervous system; DCs: dendritic cells; EAE: experimental autoimmune encephalomyelitis; IL: interleukin; INF: interferon; IR: infra-red; LFB: Luxol Fast Blue; Mal-MSA: maleylated murine serum albumin; MS: Multiple sclerosis; ox-LDL: oxidized-low-density lipoprotein; PFA: paraformaldehyde; PRRs: pattern recognition receptors; SRA: scavenger receptor A; TAU: Tel Aviv University; TNF: tumor necrosis factor; WT: wild-type.

## Competing interests

The authors declare that they have no competing interests with the contents in this paper.

## Authors’ contributions

HLB,and DF designed research, analyzed the results and wrote the paper. HLB performed the experiments. All authors read and approved the final manuscript.

## Supplementary Material

Additional file 1 ** Figure S1.Cytokine profile of CD4+ T-cells from naïve WT and SRA**^**−/−**^**.** Splenocytes were isolated from naive C57BL/6 and SRA^−/−^ mice. CD4^+^ T cells were positively enriched using magnetic beads according to the suggested protocol (551539; BD, Franklin Lakes, NJ). CD4 ^+^ T‒cells were plated in 96‒well round‒bottom plates at concentration of 3 × 10^5^ and stimulated with anti CD3 antibody in a serum free medium. After 40 hrs, the cell supernatant was collected for cytokine measurementwas done by ELISA. Click here for file
