# Supplementary figures and images for: Expression of Scavenger receptor A on antigen presenting cells is important for CD4+ T-cells proliferation in EAE mouse model
Source: J Neuroinflammation. 2012 Jun 7;9:120. doi: 10.1186/1742-2094-9-120 (PMC3466445; doi:10.1186/1742-2094-9-120)

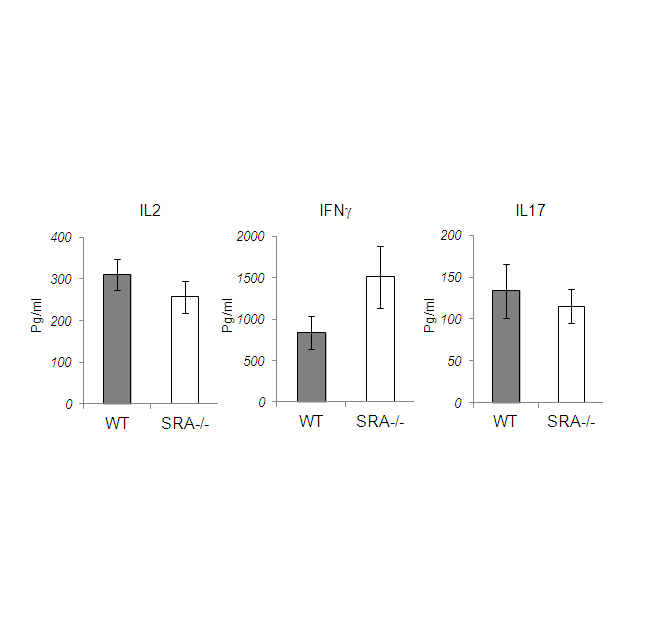

Supplement: Additional file 1 — Figure S1.Cytokine profile of CD4+ T-cells from naïve WT and SRA−/−. Splenocytes were isolated from naive C57BL/6 and SRA−/− mice. CD4+ T cells were positively enriched using magnetic beads according to the suggested protocol (551539; BD, Franklin Lakes, NJ). CD4 + T‒cells were plated in 96‒well round‒bottom plates at concentration of 3 × 10^5^ and stimulated with anti CD3 antibody in a serum free medium. After 40 hrs, the cell supernatant was collected for cytokine measurementwas done by ELISA. [file 1742-2094-9-120-S1.tiff]
